# Accelerated Solvent Extraction and Pulsed Electric Fields for Valorization of Rainbow Trout (*Oncorhynchus mykiss*) and Sole (*Dover sole*) By-Products: Protein Content, Molecular Weight Distribution and Antioxidant Potential of the Extracts

**DOI:** 10.3390/md19040207

**Published:** 2021-04-07

**Authors:** Min Wang, Jianjun Zhou, Maria Carmen Collado, Francisco J. Barba

**Affiliations:** 1 Nutrition and Food Science Area, Preventive Medicine and Public Health, Food Science, Toxicology and Forensic Medicine Department, Faculty of Pharmacy, Universitat de València, Avda. Vicent Andrés Estellés, s/n, 46100 Burjassot, Valencia, Spain; minwang@alumni.uv.es (M.W.); jianz@alumni.uv.es (J.Z.); 2 Department of Biotechnology, Institute of Agrochemistry and Food Technology-National Research Council (IATA-CSIC), Agustin Escardino 7, 46980 Paterna, Valencia, Spain; mcolam@iata.csic.es

**Keywords:** ASE, PEF, fish by-products, protein, SDS-PAGE, antioxidant

## Abstract

Fishery by-products are rich in biologically active substances and the use of green and efficient extraction methods to recover these high-added-value compounds is of particular importance. In this study, head, skin and viscera of rainbow trout and sole were used as the target matrices and accelerated solvent extraction (ASE) (45–55 °C, 15 min, pH 5.2–6.8, 103.4 bars) and pulsed electric fields (PEF) (1–3 kV/cm, 123–300 kJ/kg, 15–24 h) were applied as extraction technologies. The results showed that ASE and PEF significantly increased the protein extract efficiency of the fish by-products (*p* < 0.05) by up to 80%. SDS-PAGE results showed that ASE and PEF treatments changed the molecular size distribution of the protein in the extracts, which was specifically expressed as the change in the area or number of bands between 5 and 250 kDa. The antioxidant capacity of the extracts was evaluated by oxygen radical absorbance capacity (ORAC) and total antioxidant capacity (ABTS) assays. The results showed that both ASE and PEF treatments significantly increased the antioxidant capacity of rainbow trout and sole skin and head extracts (*p* < 0.05). ASE and PEF extraction processes can be used as new technologies to extract high-added-value compounds from fish by-products.

## 1. Introduction

According to the Food and Agriculture Organization of the United Nations report, global fish production increased from 19 million tons in 1950 to 178.5 million tons in 2018, about a nine-fold increase; per capita, consumption of fish products has increased from 9 kg in 1961 to 20.5 kg in 2018, an increase of about 2.2 times [1,2].

At the same time, in the manufacturing process of fish products, a large number of by-products are also produced, including viscera, skin, bones, fins, heads, etc., and these by-products account for 30~70% of the total weight of the fish [3,4,5]. Fish by-products have attracted a growing interest over recent years due to society’s awareness regarding the sustainable use of resources and the search for alternative nutrient sources. Fish by-products have healthy nutritional and bioactive compounds (such as protein and bioactive peptides, polyunsaturated fatty acids, etc.) [1,4,6,7].

In the past, due to the dual constraints of technology and knowledge, fish by-products were usually directly discarded. The spoilage of the by-products created a great burden on the environment, such as nourishing microorganisms, promoting the release of harmful gases and polluting land and water [8,9].

In recent years, researchers have focus on the development of high nutritional value components in fish by-products, including collagen, phospholipids and fish oil [3,4,5]. Studies have shown that these high-added-value compounds play an important role in human health. For example, some researchers have shown that collagen in fish skin is a natural ingredient, which can promote skin cell regeneration and metabolism and improve skin elasticity, with long-term consumption of fish collagen leading to a delay in skin aging and reduction of the formation of wrinkles and stains in human skin [10,11,12]. The phospholipids found in the fish head are an important source of neurotransmitter synthesis in the human brain, which can enhance human memory, thinking and analysis capabilities, and can control the degeneration of brain cells and delay aging [13,14]. Moreover, fish oil is rich in docosahexaenoic (DHA) and eicosapentaenoic (EPA) acids, which can help reducing the risk of cardiovascular and cerebrovascular diseases, such as atherosclerosis, heart disease, high blood pressure and other diseases [15,16,17].

During the process of the extraction of high-added-value compounds from fish by-products, the extraction efficiency, green conditions and the impact of labile nutrients and bioactive compounds must be considered. Accelerated solvent extraction (ASE) has been used in the food industry as an effective extraction method. For example, ASE has been used to extract natural compounds with antioxidants and anti-inflammatory properties from Passiflora species, seaweed and other plants [18,19,20]. In these studies, ASE not only shortened the extraction time, but also preserved the activity of natural products. In fishery-related research, ASE has been used to evaluate polychlorinated biphenyls, polybrominated diphenyl ethers and organochlorines in fish samples [21,22,23]. However, the available literature regarding the application of ASE to obtain nutrients and bioactive compounds from fish by-products is scarce.

Meanwhile, pulsed electric fields (PEF), as a short-term electrical pulse effect, can keep the thermal effect at a low level and retain the flavor and quality of the food to a great extent. The use of PEF-assisted extraction will only destroy the biological cells in the food matrix without harmful effects on the food, and at the same time, increase the extraction rate of juice and valuable compounds [24,25,26,27]. PEF has also been used in aquatic products. For example, PEF has been used to extract bioactive compounds from fish bones and has also been evaluated the potential of PEF for obtaining antioxidant compounds from fish residues [28].

Taking into account the large consumption demand for rainbow trout and sole and the high-value nutrients in their by-products, and PEF and ASE being less used in the recovery of these two fish by-products, this study uses ASE and PEF to extract its bioactive compounds. Further analyzed are the protein content, protein molecular distribution and antioxidant capacity of the extract.

## 2. Results and Discussion

### 2.1. Protein and Moisture Content in Rainbow Trout and Sole By-Products

The total protein and moisture content of all rainbow trout and sole by-products are shown in Table 1. As can be seen in the table, the protein content of rainbow trout skin is higher than that of head and viscera, and the wet basis accounts for close to 21% (*w/w*). The sole viscera had the highest protein content (≈21%). In terms of protein content, rainbow trout and sole by-products have high recycling value. The water content of the two fish by-products is relatively close, approximately distributed between 60 and ~70% (*w/w*).

### 2.2. Protein Extraction Efficiency

**Figure 1:**
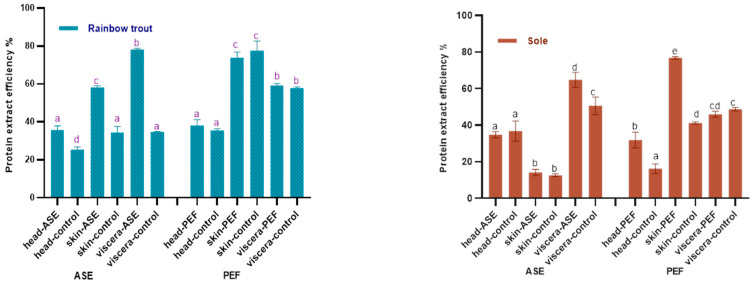
Protein content in control and optimal accelerated solvent extraction (ASE)/pulsed electric fields (PEF) extracts from by-products of Rainbow trout and Sole (head, skin, viscera). Results are expressed as mean ± standard deviation. Different letters (a–d) in the bars indicate statistically significant differences (*p* < 0.05) for each treatment (ASE or PEF).

Figure 1 shows the effects of ASE and PEF on the protein extraction rate of rainbow trout and sole, respectively. With regard to the ASE extraction groups, for rainbow trout, the highest protein extraction rate is the viscera group, which is close to 80%, followed by skin and head samples. The application of ASE significantly increased the protein extraction rate of each rainbow trout side stream (*p* < 0.05). As for the sole, the highest protein extraction efficiency was obtained for viscera, close to 60%, followed by head and skin. This is related to the different skin tissue structures of the two fishes. Compared with rainbow trout skin samples, the texture of sole skin is harder after freeze-drying, and it is more difficult to grind and mix well with diatomaceous earth, which results in a lower protein extraction rate. It can be also seen that the application of ASE significantly increased the protein extraction rate from sole viscera (*p* < 0.05), but the protein extraction rate from head and skin did not increase significantly (*p* > 0.05).

The results also showed that after using PEF, the protein extraction efficiency of both two fish skins is higher—nearly 80%—followed by the viscera and head. From a sample point of view, the protein content in fish skin is as high as 70%, which is higher than head and viscera. After PEF treatment, the sole skin samples were soaked in water for 24 h and continuously whipped. Compared with the sole skin samples of the ASE group, the hard sole skin fragments were mixed with diatomaceous earth, this process being better in promoting the extraction of protein from fish skin and thus improving the efficiency of protein extraction from fish skin. Interestingly, compared with the control group, PEF only significantly increased the protein extraction rate of sole skin and head (*p* < 0.05), but did not significantly increase the protein extraction rate of by-products in rainbow trout (*p* > 0.05).

In recent years, there has been relatively abundant research on the extraction of protein from fish by-products. Among the extraction of fish by-products protein, there are relatively many studies on collagen. For instance, a review by Ahmed et al. summarized the application research of different extraction methods in the extraction of collagen from the by-products of different fish [29]. Veeruraj et al. used acid extraction and pepsin extraction to extract 80% and 7.1% of collagen (dry basis) from ocean eel skin, respectively. In their study, the acid extraction method obtained higher protein extract efficiency in eel skin, which was similar to the PEF and higher than the ASE extract efficiency in our study [30]. However, in terms of extraction time, the process of soaking fish skin in the acid extraction method takes three days, which increased a lot extraction time compared with our study and will increase the cost of actual industrial production. Similarly, Yu et al. used the response surface method to study the effect of extraction parameters on the extraction of collagen from the skin of large yellow croaker. When the pepsin concentration was 1389 U/g, the solid–liquid ratio was 1:57 and the hydrolysis time was 8.67 h, the extraction rate of collagen reached 84.85% [31]. Similar to this study, the new extraction technology has been also applied in the extraction of fish by-product crude protein. Approximately 40% by weight in mackerel is regarded as a by-product, and studies have shown that as a new extraction technology like ASE and PEF, the ultrasound-assisted method has achieved good results in the protein extraction of mackerel by-products. Carlos et al. used the ultrasonic-assisted acid/alkali method to increase the yield of protein. Their research results showed that ultrasonic-assisted sequential acid and ultrasonic-assisted alkaline extraction can obtain almost 100% and 95% of the protein in mackerel by-products, respectively (ultrasound-assisted extraction time approximately 75 min). Compared with this study, although the protein extraction rate in this study was less than 95%, this study chose water as the extraction reagent, which reduced the cost of the reagent, and the ASE and PEF treatment time was less than 75 min, which greatly shortened the extraction time [32].

From the principle of ASE, the strong interaction force between the solute and the matrix caused by the van der Waals force, hydrogen bonds, the dipole attraction of the solute molecules and the active site of the sample matrix can be greatly reduced under high temperature and pressure. This accelerates the analytical kinetics process of the solute molecule, reduces the activation energy required for the analytical process, reduces the viscosity of the solvent, thus reducing the blocking of the solvent entering the sample matrix, and increases the diffusion of the solvent into the sample matrix [23].

Unlike ASE, PEF is one of the processing technologies based on electricity [26]. The application of short electrical pulses at high voltages can keep the control of thermal effects at a low level, making it different from thermoelectric technologies such as ohmic heating [33]. These properties make PEF a promising technology, able to destroy the biological cells in the food matrix without any harmful effects on the properties of the food [34]. Studies have shown that short pulse electric fields (μs to ms) in the range of 100–300 V/cm to 20–80 kV/cm applied by PEF can cause cell membranes to disintegrate and form membrane pores (temporary or permanent) [26,34]. Zhou et al. studied the extraction effect of PEF technology to obtain protein from mussels. In the case of PEF (triangular pulse power waveform and pulse duration are 2 μs) and under the best estimation conditions (electric field strength is 20 kV/cm, pulse number 8, enzymatic hydrolysis 2 h), the maximum yield of protein extracted can reach 77.08% [35]. In our study, the protein content of the sole head and skin samples increased significantly under the PEF treatment, which may be due to the phenomenon of “electroporation”, which promoted the dissolution of protein in the cells.

### 2.3. Protein Molecular Weight Distribution in Fish By-Product Extracts

**Figure 2:**
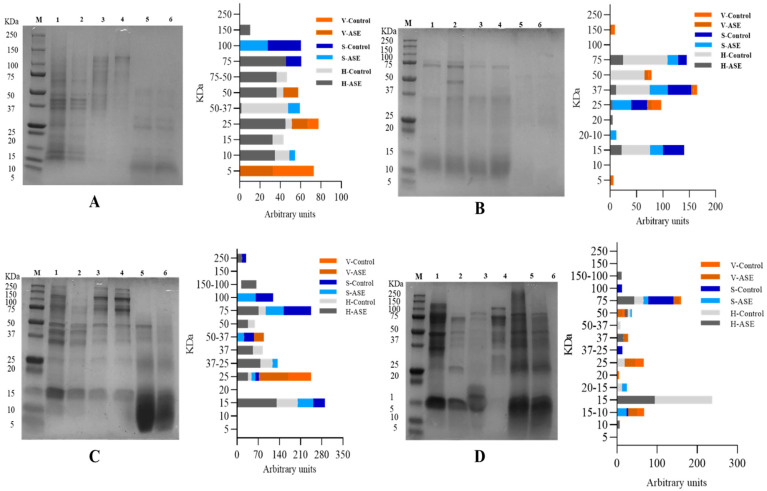
SDS-PAGE. (**A**): Rainbow trout, Accelerated Solvent Extraction (ASE); (**B**): Rainbow trout, Pulsed Electric Fields (PEF); (**C**): Sole, Accelerated Solvent Extraction (ASE); (**D**): Sole, Pulsed Electric Fields (PEF). Corresponding to 1–6 are Head—ASE/PEF, Head—Control, Skin—ASE/PEF, Skin—Control, Viscera—ASE/PEF and Viscera—Control. M means molecular weight standard.

The SDS-PAGE bands of rainbow trout and sole side stream extracts are shown in Figure 2. For rainbow trout, after ASE treatment, the protein molecular weights in the fish head, skin and viscera extract are respectively distributed in 150–10 kDa, 100–10 kDa and 50–5 kDa; the corresponding control group corresponds to the distribution in 75–10 kDa, 100–75 kDa and 25–5 kDa. This shows that ASE makes the protein types in the rainbow trout side stream extract more abundant. The difference is that the protein distribution of the PEF treatment group and PEF control group is the same. The molecular weights of proteins extracted from head, skin and viscera are distributed in 75–15 kDa, 75–15 kDa and 150–5 kDa, respectively. For sole, after ASE extraction, the protein molecular weights in head, skin and viscera extracts were distributed in 250–15 kDa, 250–15 kDa and 50–25 kDa, respectively; the corresponding control group results were 75–15 kDa, 100–15 kDa and 25 kDa. This also shows that ASE increased the protein abundance in the sole side stream extract. The PEF treatment group only increased the protein of the fish head group at the molecular weight of 150–100 kDa.

In order to compare the amount of protein extracted at different molecular weights more directly, ImageJ was used to calculate the area of each band. The results of the by-products’ protein distribution from rainbow trout–ASE and PEF, and sole–ASE and PEF are also shown in Figure 2. It can be seen from Figure 2A that ASE promoted the extraction of proteins with molecular weights of 150 kDa and 75 kDa from rainbow trout heads, which does not occur in the control group. At the same time, from the results of the percentage of the strip area, ASE extraction increased the protein content of 75–50 kDa, 25 kDa, 15 kDa and 10 kDa in the rainbow trout head group. The distribution of protein bands in rainbow trout skin and viscera is relatively simple. ASE extraction increased the protein distribution at 50–37 kDa and 10 kDa in the skin group and the protein with a molecular weight of 50 kDa in the viscera group. It is not difficult to see that the ASE extraction method increased the abundance of proteins extracted from rainbow trout by-products. However, the effect of PEF on the protein band distribution of rainbow trout by-products is not consistent. Specifically, it can be seen from Figure 2B that the proportion of proteins at 75 kDa, 37 kDa and 15 kDa in the rainbow trout head samples treated with PEF decreased, and only the control group appeared with 50 kDa molecular weight proteins. In the fish skin group, although PEF extracted new proteins with a molecular weight of 20–10 kDa, the proportion of proteins at 37 kDa and 15 kDa was lower than that of the control group, and the proportion decreased by more than 10%. The band distribution of PEF in the viscera group is consistent with the control group. However, the protein ratio at 37 kDa and 25 kDa in the PEF group is nearly 1 time lower than that in the control group. This indicates that PEF failed to promote the extraction of different molecular weight proteins in rainbow trout by-products, which is consistent with the results of the protein extraction rate.

For sole, Figure 2C shows that ASE promoted the extraction of proteins with molecular weights of 250 kDa and 150–100 kDa from sole heads, which did not occur in the control group. From the perspective of the relative area ratio of the bands, ASE extraction greatly increased the area ratio of the protein bands at 75 kDa, 50 kDa, 37 kDa, 37–25 kDa, 25 kDa and 15 kDa in the sole head sample. Compared with the skin control group, the band area ratio of the ASE extraction group at 100 kDa, 75 kDa, 50–37 kDa, 37 kDa decreased, and the protein ratio at 37–25 kDa and 15 kDa increased, which shows that the ASE can promote extraction of small molecular weight proteins from sole skin. Similarly, ASE increased the ratio of 50–37 kDa and 25 kDa molecular weight proteins in viscera. Consistent with rainbow trout, ASE promoted the extraction of proteins of different molecular weights in the sole by-products and obtained some band distributions that did not exist in the control group. The results showed that PEF (Figure 2D) mainly promoted the extraction of protein from sole viscera, manifested by increasing the protein content of 75 kDa, 50 kDa, 37 kDa, 20 kDa and 15–10 kDa. After sole fish head was treated with PEF, the proteins with molecular weights of 150–100 kDa, 75 kDa and 50 kDa increased, and the proteins at 50–37 kDa, 25 kDa, 20–15 kDa and 15 kDa decreased. This shows that PEF treatment increases the extraction of large molecular weight proteins in sole head samples and inhibits the dissolution of small molecular weight proteins. In contrast, after sole skin was treated with PEF, the protein of 20–15 kDa and 15–10 kDa increased, and the ratio of protein of 100 kDa, 75 kDa and 37–25 kDa decreased. This shows that PEF promotes the extraction of small molecular weight proteins in fish skin and is not conducive to the dissolution of large molecular weight proteins in sole skin. There have been research reports on analyzing the peptide distribution of fish skin. Gokula et al. extracted 19.27 ± 0.05 mg/g collagen from sole skin under the conditions of acetic acid concentration of 0.54 M, sodium chloride concentration of 1.90 M, a liquid-to-solid ratio of 8.97 mL/g and 32.32 h, and SDS-PAGE analysis revealed that the molecular weight of collagen peptides was between 118 kDa and 116 kDa [32]. The results of this study showed that the ASE group extracted 100 kDa protein from sole skin, while the PEF group lacked the band distribution at 100 kDa. This shows that different extraction methods differ in the extraction of the same type of fish skin.

**Figure 3:**
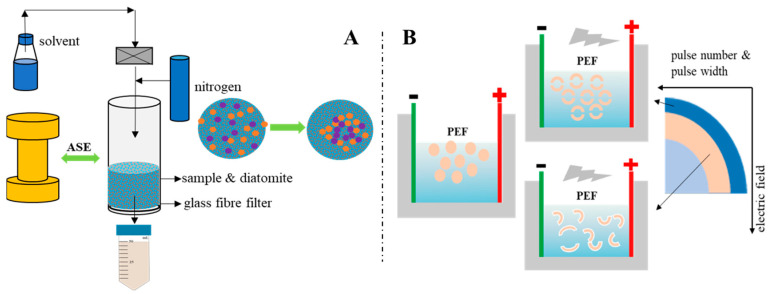
Schematic diagram of the (**A**) accelerated solvent extraction (ASE) and (**B**) pulsed electric fields (PEF) effect, respectively.

As for ASE, pressure extraction is an important factor in changing the protein content and properties of the extract. Gómez-Guillén et al. used high pressures (250–400 MPa) to treat fish skin and found that high pressure not only increased the yield of fish skin collagen, but also changed the molecular weight distribution of fish skin protein, showing that a high-pressure extraction method is superior to traditional methods in fish skin collagen extraction [36]. The pressure in the extraction of fish side stream protein in our ASE methodology was nearly 10 MPa, which is lower than 250–400 MPa, but the result still shows that pressure changes the molecular weight of fish side stream protein, which is conducive to extracting more low-molecular-weight proteins from fish by-products. Related studies have confirmed that pressurization will have a certain impact on the structure of protein molecules in food. For example, a pressure lower than 150 MPa can affect the quaternary structure of a protein, 200 MPa can affect the tertiary structure and 300–700 MPa can change the secondary structure [37,38]. The pressure in this study was only close to 10 MPa, which may affect the spatial conception of protein molecules. Relevant studies have shown that during the pressurization process, the pressure may lead to the destruction of non-covalent interactions and changes in intermolecular and intramolecular and solvent–protein interactions, thereby changing the natural conformation of proteins [39]. Normally, high pressure will not affect the covalent bonds of protein molecules and will not destroy the peptide bond structure in protein molecules [40]. From the perspective of the working principle of the ASE method, at a certain temperature, pressurization can increase the permeability of the solvent, making it easier to enter the sample matrix and increase the contact time between the sample and the solvent [41]. The related effect is shown in Figure 3. Therefore, it can be inferred that the change in SDS-PAGE results is due to ASE changing the solubility of different molecular weight proteins in fish by-products in water. ASE extraction not only increased the total protein content in the extract; the SDS-PAGE results further showed that ASE also promoted the increase in the content of certain specific molecular weight proteins, which is meaningful for obtaining specific molecular weight proteins from fish by-products.

For PEF, the specific mechanism of protein distribution changes caused by PEF has not been accurately determined. However, related studies have proved that protein molecules will be polarized at low PEF intensity, and their hydrophobic amino acids will gradually be exposed to the solvent as the electric field intensity increases. The final unfolded protein may become an aggregate of weak covalent and non-covalent bonds under relatively high field strength [42]. After a certain PEF intensity is exceeded, the thermal effect caused by the arc will cause the denaturation and aggregation of heat-sensitive proteins [43]. Studies have also shown that PEF can destroy the secondary structure of proteins, such as increasing the ratio of β-sheets and reducing the content of α-helices [44,45]. Since no relevant studies have been found to show that PEF can cause the breakage of the primary structure of the protein–peptide bond, the changes in the distribution of protein bands in this study are mainly related to two aspects. One is that PEF causes cell breakage in fish by-products to accelerate protein dissolution (Figure 3); the other is that the PEF electric field causes the exposure of protein hydrophobic amino acids and further protein aggregation occurs, which may cause some protein molecular weight changes during the extraction process.

### 2.4. Antioxidant Capacity

#### 2.4.1. Oxygen Radical Absorbance Capacity (ORAC)

**Figure 4:**
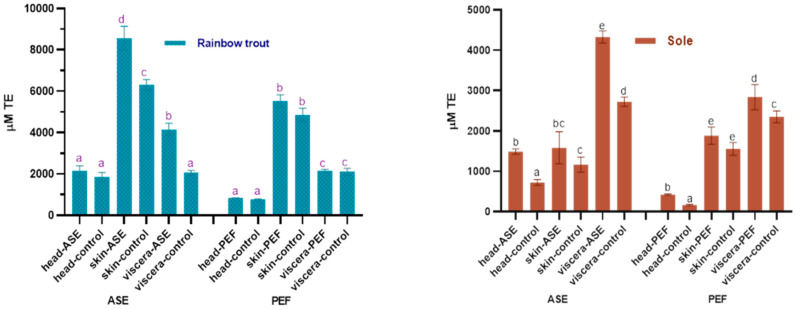
Total antioxidant capacity (ORAC) in control and optimal ASE/PEF extracts from by-products (head, skin and viscera) of Rainbow trout and Sole. ORAC: Oxygen radical absorbance capacity; ASE: accelerated solvent extraction; PEF: pulsed electric fields. Results are expressed as mean ± standard deviation. Different letters in the bars indicate statistically significant differences (*p* < 0.05).

**Figure 5:**
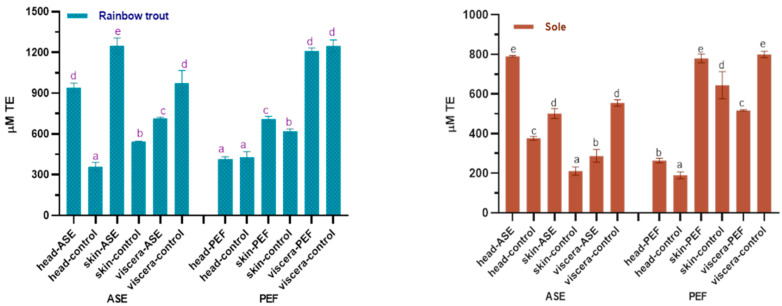
Total antioxidant capacity (ABTS) in control and optimal ASE/PEF extracts from by-products (head, skin and viscera) from Rainbow trout and Sole. ABTS: Trolox equivalent antioxidant capacity; ASE: accelerated solvent ex-traction; PEF: pulsed electric fields. Results are expressed as mean ± standard deviation. Different letters in the bars indicate statistically significant differences (*p* < 0.05).

The antioxidant properties in the extracts are worthy of attention. In this study, the oxygen radical absorbance capacity (ORAC) and total antioxidant capacity (ABTS+) scavenging ability of the fish by-product extracts were used to judge the antioxidant capacity of the bioactive compounds in the extracts, and the results are shown in Figure 4 and Figure 5. For rainbow trout by-product extracts, whether in the ASE or PEF group, skin extract has the strongest oxygen radical absorbance capacity, followed by viscera and head. From Figure 4, ASE significantly increased the oxygen radical absorbance capacity of rainbow trout skin and viscera extracts (*p* < 0.05), while PEF had no significant effect on its oxygen radical absorbance capacity (*p* > 0.05). For sole, the visceral anti-oxygen free radicals in ASE and PEF extract products are the strongest, followed by skin and head. ASE extraction significantly increased the oxygen radical absorbance capacity of sole head and viscera (*p* < 0.05), and slightly increased the antioxidant capacity of sole skin extracts (*p* > 0.05), and PEF significantly increased the oxygen radical absorbance capacity of head and viscera (*p* < 0.05), and slightly increased the anti-oxygen free radical ability of skin (*p* > 0.05).

#### 2.4.2. ABTS+ Scavenging Ability

Figure 5 shows the effects of ASE and PEF extraction on the ABTS+ scavenging ability of two fish by-products. The results showed that ASE significantly increased the anti-ABTS+ ability of rainbow trout and sole head and skin extracts (*p* < 0.05), but meanwhile significantly reduced the anti-ABTS+ ability of the two fish viscera extracts (*p* < 0.05).

The application of PEF technology significantly increased the ability of rainbow trout skin extract to resist ABTS+ (*p* < 0.05) and did not affect the head and viscera significantly (*p* > 0.05). Unlike rainbow trout, the use of PEF significantly increased the ABTS+ scavenging capacity of sole head and skin extract (*p* < 0.05), but significantly reduced the antioxidant capacity of viscera extract (*p* < 0.05). From the results of the ORAC and ABTS experiments, both ASE and PEF increased the Trolox equivalent value of rainbow trout and sole skin and head extracts, indicating that the antioxidant properties of the corresponding extracts were enhanced. However, the calculation results of ASE and PEF on the antioxidant capacity of visceral extracts are inconsistent. Both ORAC and ABTS can characterize the antioxidant activity of active substances, but the principles of the two are different. The principle of the ORAC experiment is to observe the ability of Trolox or the antioxidants in the extract to resist 2,2’-azobis (2-amidinopropane) dihydrochloride (AAPH) to reduce the fluorescence intensity [46], while that of the ABTS method is to measure the ability of the extracts or Trolox to eliminate ABTS+ [47]. Although the sources of free radicals corresponding to the two methods are inconsistent, and the final Trolox quantification is not completely consistent, the results still show that both ASE and PEF treatments increase the antioxidant activity of the extract.

## 3. Materials and Methods

### 3.1. Samples

The experimental rainbow trout and sole samples were obtained from a local supermarket. Fish samples were dissected in the laboratory to obtain skin, head and viscera. The sample for the ASE extraction experiment needs to be freeze-dried under vacuum at −48 °C for 72 h, then the sample is crushed into powder and stored at −20 °C for later use. The experimental samples of PEF were freshly dissected fish by-products.

### 3.2. Chemicals and Reagents

AAPH (2,2′-azobis-2-methyl-propanimidamide)(), Trolox (6-hydroxy-2,5,7,8-tetramethylchroman-2-carboxylic acid), fluorescein sodium salt and potassium persulfate (K_2_S_2_O_8_) were purchased from Sigma-Aldrich (Steinheim, Baden-Württemberg, Germany). Sodium dodecyl sulphate-polyacrylamide gel electrophoresis (SDS-PAGE)-related reagents were purchased from Bio-Rad. Diatomaceous earth and other materials for the generation of ASE^®^ extracts were purchased from Dionex (Dionex, Leeds, UK).

### 3.3. Extraction Technologies

#### 3.3.1. ASE Extraction

The ASE extraction conditions were selected based on some experimental work carried out in the laboratory with sea bass samples [48]. According to those experiments, the mix ratios of fish head, viscera, skin and diatomaceous earth were 1.0:2.0, 1.5:3.0 and 2.0:2.0 (g/g), respectively. Fish by-products and sample and diatomaceous earth were thoroughly mixed in a mortar and placed into the extraction tank and the ASE extraction conditions were settled using a pressure of 103.4 bars. For these experiments, an ASE-200 Accelerated Solvent Extractor (Sunnyvale, CA, USA) was used. The standard operating conditions were as follows: preheating period (1 min), heating period (5 min), flush volume (60%), nitrogen purge (60 s), and extraction pressure (103.4 bars). More detailed extraction conditions are shown in Table 2. After the ASE extraction was completed, the extracts were collected in extraction bottles and kept at −20 °C until needed for analyses. At the sample time, the control experiment was set up. Deionized water was used as the extraction reagent at normal pressure to ensure that the extraction time, pH and temperature were consistent with the ASE experimental group. After the extraction was completed, the extract was filtered using filter paper, and the obtained sample was stored at −20 °C for later use.

**Figure 6:**
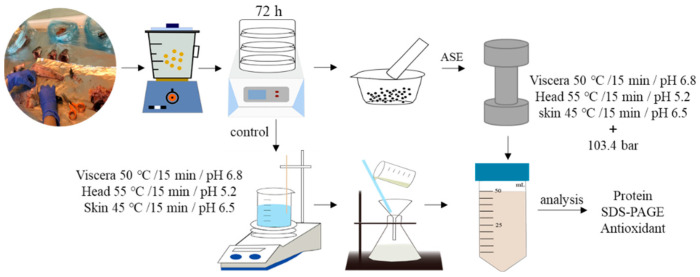
The accelerated solvent extraction (ASE) processes followed for the recovery of high-added-value compounds from rainbow trout and sole by-products.

**Figure 7:**
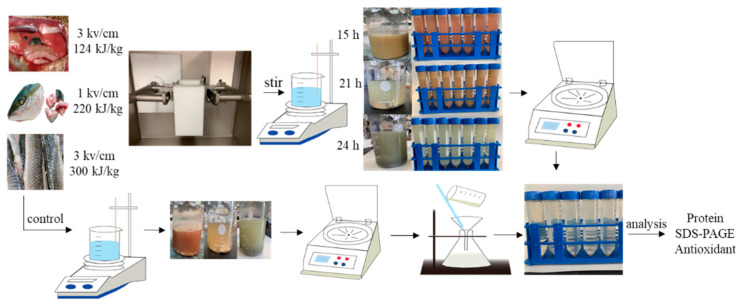
The pulsed electric fields (PEF) extraction processes followed for the recovery of high-added-value compounds from rainbow trout and sole by-products.

Figure 6 and Figure 7 show the ASE and PEF extraction processes followed for the recovery of high-added-value compounds from rainbow trout and sole by-products, respectively.

#### 3.3.2. PEF Extraction

Similarly, PEF optimal extraction conditions were previously selected at the laboratory (data not shown) using a PEF-Cellcrack III (German Institute of Food Technologies (DIL)) equipment (ELEA, Quakenbrück, Osnabrück, Germany). Specifically, the fresh fish by-products (head, skin and internal organs) were placed in the processing chamber, and a certain amount of tap water was added. The conductivity was maintained between 1000 and ~2000 µs/cm. Then, the samples were PEF-processed according to the experimental conditions (Table 1). The processed samples were introduced into a beaker and were continuously stirred using a magnetic stirrer at room temperature. Finally, the obtained extracts were centrifuged at 4000× rpm at 4 °C for 15 min and the supernatant was taken and filtered to obtain the sample.

### 3.4. Chemical Analyses

#### 3.4.1. Proximate Composition

Firstly, the moisture and protein content of the fish by-product samples were tested. Moisture was determined by oven-drying until constant weight at 103 ± 2 °C [49]. Protein was determined according to the Kjeldahl method [50] (see Section 3.4.2.).

#### 3.4.2. Protein Content

The protein content was determined according to the Kjeldahl method [50]. Five grams of zeolite, 3 g of potassium sulphate, 5 drops of copper sulphate solution and 2 mL sample were added into the nitrification tube, then 5 mL of 98% concentrated sulphuric acid were added and the mixture was heated at 120 °C until the solution was clear and translucent. The automatic Kjeldahl analyzer was used to convert the ammonia ions in the sample into ammonia gas, and boric acid was used as the absorption liquid. Finally, 0.1 M hydrochloric acid was used as the titration reagent, and methyl orange as the end point indicator of the titration. The calculation formula of nitrogen content was:

(1) $$\mathrm{Nitrogen}\;\%=\frac{\left[\left(\mathrm{mL}\;\mathrm{standard}\;\mathrm{acid}-\mathrm{mL}\;\mathrm{blank}\right)*N\;\mathrm{of}\;\mathrm{acid}*1.400\right]}{\left(\mathrm{Weight}\;\mathrm{of}\;\mathrm{sample}\;\mathrm{in}\;\mathrm{grams}\right)}$$

The calculation formula of protein content is:

(2) Protein %=6.5*Nitrogen %

#### 3.4.3. Molecular Size Distribution (SDS-PAGE)

The SDS-PAGE method refers to the relevant literature and is slightly modified [48]. Prior to the SDS-PAGE experiment, the sample buffer was prepared. Five hundred milligrams of SDS, 2.46 g of Tris-HCl, 6.25 mL of glycerol, 2.5 mL of bromophenol blue reagent and 13 mL of deionized water were introduced into a beaker and mixed on a magnetic stirrer. Then, the pH was adjusted to 6.8 with the diluting of a sodium hydroxide solution, then deionized water was added up to 25 mL. Eight milligrams of dithiothreitol (DTT) were added to 500 μL of the sample buffer, then the mixture was stored under darkness and marked as “A”.

In order to configure the electrophoresis strip fixing solution, 400 mL of methanol, 100 mL of acetic acid and 500 mL of water were mixed. Two hundred milliliters of methanol, 100 mL of acetone and 700 mL of deionized water as the decolorizing solution were mixed. Then, the electrophoresis solution was prepared, that is, 0.5 g SDS, 7.2 g glycine and 1.515 g Trizma base were weighed and then dissolved in 1000 mL of distilled water.

To prepare the sample, 100 µL of extracts and 400 µL of acetone were mixed, vortexed for 10 s, and centrifuged at 11,000 rpm at 4 °C for 10 min. The supernatant was removed and placed in a fume hood for 5 min, then the remaining acetone reagent was volatilized. The precipitate was then rinsed with 100 µL of deionized water and sonicated at 25 °C for 20 s to completely dissolve the precipitate, named “B”. Then A and B were mixed (*v/v*, 20 μL/20 μL) and heated at 95 °C for 5 min.

Bio-Rad equipment was used for gel electrophoresis. The label and sample loading volumes were 10 μL and 25 μL, respectively. The equipment was maintained under a constant voltage of 80 V, and when the marker band reached the bottom of the gel, the electrophoresis experiment was finished. Then, the gel was removed and the fixative was added to soak for 30 min. Next, the fixative was removed and Coomassie brilliant blue dye was added for 30 min. Finally, the decolorizing solution was added and was kept shaking for 24 h until the protein bars in the gel were clear.

#### 3.4.4. Total Antioxidant Capacity

##### Oxygen Radical Absorbance Capacity Test

The oxygen radical absorption capacity (ORAC) test is used to evaluate the oxygen radical absorption capacity of the sample. The fluorimetric method described by Barba et al. was applied [51]. We added 50 µL of phosphate buffer (pH 7.0~7.4), 1 mM Trolox and sample to 96-well plates, then added 50 µL of fluorescein sodium salt, incubated at 37 °C for 10 min, and added 25 µL of AAPH (120 mg/mL). The absorbance value of the sample was measured at 520 nm and a total of 45 cycles were tested. Each group of samples set five holes in parallel. The test was repeated three times, and the coefficient of variation of the data was less than 15%. The calculation formula was:

(3) $$\mathrm{ORAC}\left(\mathrm{trolox}\right)=\frac{\mathrm{Asample}-\mathrm{Ablank}}{\mathrm{Atrolox}-\mathrm{Ablank}}$$

##### Trolox Equivalent Antioxidant Capacity Assay (TEAC)

The spectrophotometric method proposed by Barba et al. was used [51]. The 2,2′-azinobis-(3-ethylbenzothiazoline-6-sulphonate) (ABTS) test method referred to the relevant literature and had slight modifications. We mixed 25 mL of 7 mM ABTS with 440 µL of 140 mM potassium thiosulphate solution to obtain a working solution and stored it at room temperature in the dark for 12~16 h before use. Then, 7 mM of the working solution were diluted with 96% ethanol to keep the absorbance value between 0.700 ± 0.020. During the test, we mixed 0.1 mL of the sample or standard with 2 mL of working solution and read the absorbance value of the reaction solution at 734 nm after it had reacted in a dark room for 3 min. The standard curve was calculated with the absorbance and concentration values of the Trolox solution and the Trolox equivalent used as the antioxidant capacity of the sample. The standard curve equation is y = 0.0014x + 0.6504, R^2^ = 0.999.

### 3.5. Software and Statistics

IMAGE-J software was used for electrophoretic band analysis. GraphPad Prism (GraphPad Software Company, La Jolla, CA, USA) was used for graph rendering, and SPSS (IBM Cop., Armonk, NY, USA) was used for data significance analysis. Analysis of variance (ANOVA) and Duncan’s multiple range test were used to estimate the significance of the difference in the mean. Each repeated analysis was performed three times, and the statistical significance was estimated at 5% level (*p* < 0.05).

## 4. Conclusions

As a green and efficient extraction technology, this study shows that ASE and PEF have shown good results in the extraction of active substances from fish by-products. The treatment of ASE and PEF made the protein extraction rate of fish by-products reach 80% and changed the distribution of molecular size. In addition, after evaluating the antioxidant capacity of the extracts, it can be shown that the treatment of ASE and PEF improves the antioxidant capacity of the skin and head from sole. Both the pressurization in the ASE extraction process and the electric field in the PEF extraction are beneficial to the extraction of soluble proteins in the by-products, which not only replaces the pollution of organic reagents in traditional extraction techniques, but also retains the antioxidant properties of active substances to the greatest extent. This is in line with the requirements of modern industry and environmental development and will play a huge role in the transformation and utilization of aquatic resources in the future.

## Figures and Tables

**Table 1:** Protein and moisture content in rainbow trout and sole by-products.

	Protein (Wet Basis%)		Moisture (%)	
	Rainbow Trout	Sole	Rainbow Trout	Sole
Head	12.9 ± 0.4 ^a^	15.8 ± 1.1 ^a^	70.3 ± 0.3 ^b^	71.1 ± 1.6 ^c^
Skin	20.8 ± 0.6 ^b^	18.8 ± 2.4 ^a^	58.3 ± 0.5 ^a^	60.3 ± 0.8 ^a^
Viscera	13.4 ± 0.6 ^a^	21.7 ± 1.9 ^b^	69.2 ± 0.9 ^b^	65.6 ± 0.7 ^b^

^a–c^ Different letters in the same column indicate statistically significant differences (*p* < 0.05).

**Table 2:** Extraction conditions for recovering bioactive compounds from rainbow trout and sole by-products using accelerated solvent extraction (ASE) and pulsed electric fields (PEF).

Methodology	ASE				PEF		
Rainbow Trout/Sole	T (°C)	t (min)	pH	Pressure (bars)	Field Strength (kV/cm)	Specific Energy (kJ/kg)	t* (h)
Head—optimal	55	15	5.2	103.4	1.00	219.765	21.329
Head—control	55	15	5.2	No	No	No	21.329
Skin—optimal	45	15	6.5	103.4	3.00	300	24
Skin—control	45	15	6.5	No	No	No	24
Viscera—optimal	50	15	6.8	103.4	3.00	123.750	15.169
Viscera—control	50	15	6.8	No	No	No	15.169

t*: Time of supplementary extraction.

## Data Availability

Not applicable.
